# A sparse Bayesian factor model for the construction of gene co-expression networks from single-cell RNA sequencing count data

**DOI:** 10.1186/s12859-020-03707-y

**Published:** 2020-08-18

**Authors:** Michael Sekula, Jeremy Gaskins, Susmita Datta

**Affiliations:** 1 Department of Bioinformatics and Biostatistics, University of Louisville, Louisville, KY USA; 2 Department of Biostatistics, University of Florida, Gainesville, FL USA

**Keywords:** Co-expression, Latent factor model, Networking, RNA sequencing, Single-cell

## Abstract

**Background:**

Gene co-expression networks (GCNs) are powerful tools that enable biologists to examine associations between genes during different biological processes. With the advancement of new technologies, such as single-cell RNA sequencing (scRNA-seq), there is a need for developing novel network methods appropriate for new types of data.

**Results:**

We present a novel sparse Bayesian factor model to explore the network structure associated with genes in scRNA-seq data. Latent factors impact the gene expression values for each cell and provide flexibility to account for common features of scRNA-seq: high proportions of zero values, increased cell-to-cell variability, and overdispersion due to abnormally large expression counts. From our model, we construct a GCN by analyzing the positive and negative associations of the factors that are shared between each pair of genes.

**Conclusions:**

Simulation studies demonstrate that our methodology has high power in identifying gene-gene associations while maintaining a nominal false discovery rate. In real data analyses, our model identifies more known and predicted protein-protein interactions than other competing network models.

## Background

Deriving co-expression networks from gene expression data is a primary goal in numerous biological studies. These networks, which are commonly referred to as gene co-expression networks (GCNs), are constructed by identifying pairs of genes that have significant associations between their expression profiles across samples. Genes are represented by nodes in GCNs and co-expression values are represented by edges that connect pairs of nodes. These edges are undirected to indicate the relationships or dependencies between genes, not the underlying cause of these associations. This makes GCNs different from gene regulatory networks, which have directed edges to infer causal relationships [1]. As demonstrated in [2], genes with similar expression patterns tend to be involved in similar cellular processes and functions. Therefore, researchers are able to identify novel interactions and relationships between genes by exploring GCNs [3, 4].

Many of the statistical methods for building GCNs have been developed for analyzing data consisting of expression values averaged over bulk populations of cells, such as microarray or bulk RNA sequencing; however, advancements in technology now allow researchers to obtain expressions at the level of a single cell. By gathering information from individual cells, new opportunities to study cellular heterogeneity are presented. This is of particular interest in GCNs since mapping gene expressions across different states of cells can lead to a better understanding of the biological mechanisms behind this heterogeneity [5]. Single-cell RNA sequencing (scRNA-seq) provides new and exciting opportunities to examine biological processes at a high resolution, yet at the same time, this data presents new statistical and computational challenges (e.g., zero-inflation, high cell-to-cell variability, multimodality) that have not been previously faced with bulk sample data [6]. Therefore, network algorithms initially developed for bulk samples are often not suitable for single-cell analysis [7].

Some algorithms for network analysis in scRNA-seq data have been recently proposed, but these methods fail to outperform general methods developed for bulk sample data [8]. To that end, we present a sparse hierarchical Bayesian factor model to explore the network structure associated with genes. The latent factors in our model adjust the gene expressions for each cell to help accommodate for the zero-inflated and overdispersed attributes of scRNA-seq data, and a GCN structure is constructed by examining the shared factors between pairs of genes. We refer to our hierarchical Bayesian factor model as HBFM.

This manuscript is organized as follows. In the “Results”, we apply our method to both simulated and real data and also compare the performance of our methodology to the performance of other network methods. A brief summary of our proposed methodology is provided in the “Discussion” and we highlight our main conclusions in the “Conclusion”. Our proposed model and GCN inference is defined in the “Methods” section.

## Results

### Datasets

To demonstrate the feasibility of our methodology, we generated simulated datasets consistent with our proposed methodology structure defined in the “Methods” section. Each *Y*_*gi*_ count was sampled from Poisson (*μ*_*gi*_), with *μ*_*gi*_ modeled from Eq. (1). The *β*_*g*_ parameters were randomly sampled from Gamma(3,0.5) and the *λ*_*if*_ parameters were randomly sampled from Lognormal (0,*ϕ*_*f*_).

**Fig. 1:**
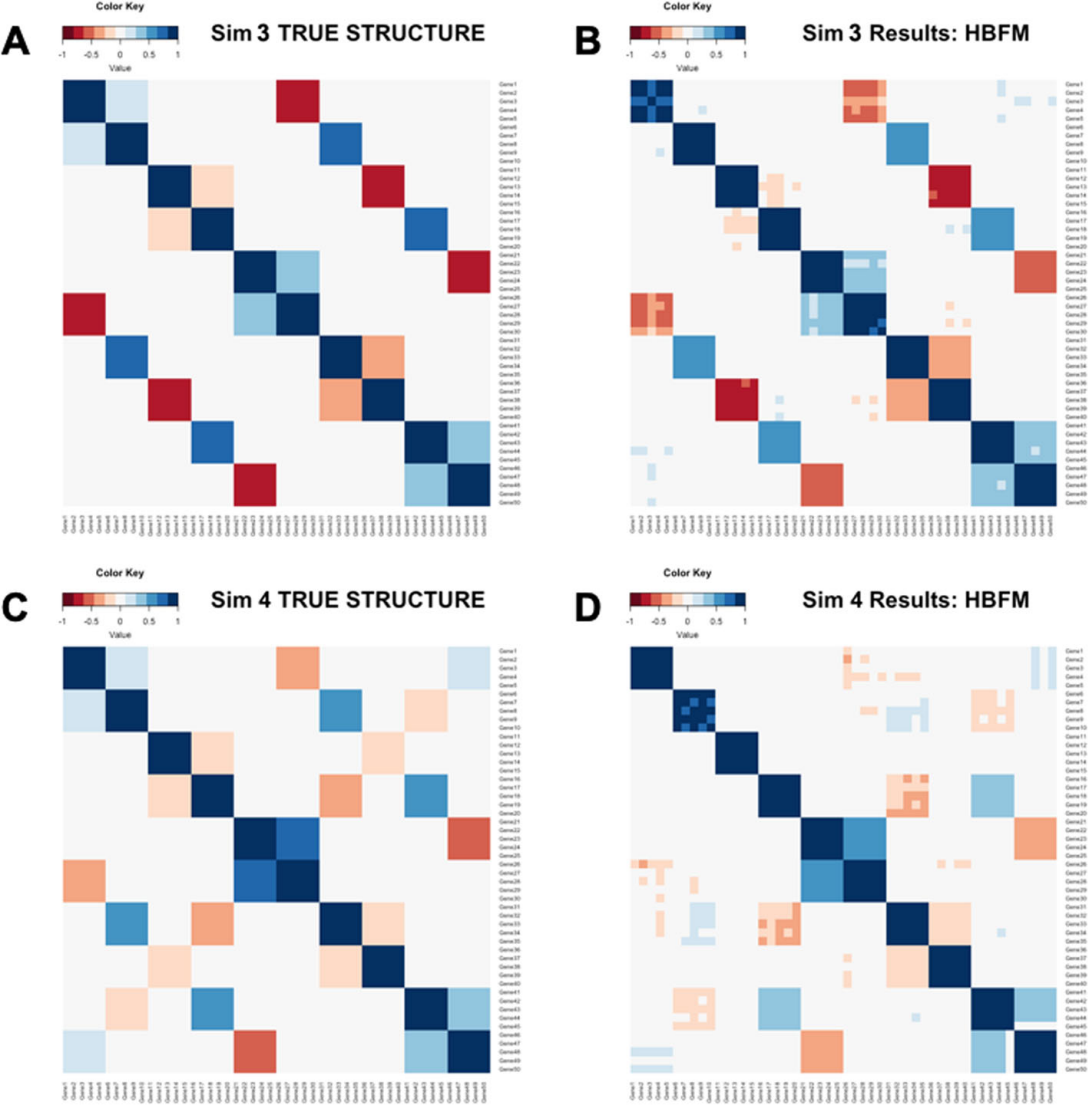
**a** Heatmap of the “true” correlation structure in Sim 3 (*F*=10,*N*=500). **b** Heatmap of the estimated correlation structure in Sim 3 by HBFM and *F*=25 factors. **c** Heatmap of the “true” correlation structure in Sim 4 (*F*=15,*N*=500). **d** Heatmap of the estimated correlation structure in Sim 4 by HBFM and *F*=25 factors

For the network structures, we fixed the values of the ***α*** matrix. In each dataset, we considered *G*=50 genes and sorted them into ten groups of five (e.g., Group 1 consisted of genes 1 - 5, Group 2 consisted of genes 6 - 10), and all genes within each factor group were assigned the same *α*_*gf*_ values. In three of the datasets, we considered the same network structure (Fig. 1a) consisting of 350 “true” edges using *F*_*sim*_=10 factors and varied the number of cells to be either *N*=125 (Sim 1), *N*=500 (Sim 3), or *N*=1,000 (Sim 5). In the other three datasets, we utilized a network structure of *F*_*sim*_=15 factors to simulate expression values, which created a network structure with 425 “true” edges (Fig. 1c). Again, the numbers of cells were set to either *N*=125 (Sim 2), *N*=500 (Sim 4), or *N*=1,000 (Sim 6). In order to define the correlation structures, the values of *ϕ*_*f*_ were fixed to be either 0.20, 0.35, 0.50, 0.65, or 0.80. In the simulations with *F*_*sim*_=10, each fixed value of *ϕ*_*f*_ was used twice (e.g., *ϕ*_1_=*ϕ*_2_=0.20, *ϕ*_3_=*ϕ*_4_=0.35) and in the simulation with *F*_*sim*_=15, each fixed value was used three times (e.g., *ϕ*_1_=*ϕ*_2_=*ϕ*_11_=0.20, *ϕ*_3_=*ϕ*_4_=*ϕ*_12_=0.35).

To evaluate the performance of our methodology on data simulated from a structure that differs from our proposed methodology, we generated count data from marginal zero-inflated negative binomial distributions via the NORmal To Anything (NORTA) algorithm [9]. The zero-inflated negative binomial distribution is a popular choice for modeling scRNA-seq count data [10–12] and the NORTA algorithm allows us to induce a “true” gene-gene correlation structure. Six datasets (Sim 7 - 12) were simulated with the same number of genes, number of cells, and network structures as the six previously described datasets (Sim 1 - 6). Therefore, the network structures for Sim 7, Sim 9, and Sim 11 have 350 “true” edges (Fig. 1a) and the network structures for Sim 8, Sim 10, and Sim 12 contain 425 “true” edges (Fig. 1c). Counts were generated with the *rnorta* function from the R package SimCorMultRes [13] and the ZIM package [14] was used to estimate the parameters of the zero-inflated negative binomial distributions from *G*=50 genes randomly selected from the 101 genes considered in our case study analysis of the mouse microglia cell (MMC) data from [15].

We also ran analyses on two real datasets to demonstrate the utility of our method on real data. The expression counts for the mouse brain single-cell (MBSC) dataset from [16] were downloaded from the Gene Expression Omnibus (GEO) database under accession number GSE60361. For this analysis, we selected the *G*=48 known and novel genetic markers displayed in Figure S6 of the supplementary materials of [16]. Cells with a library size of zero were removed, leaving a total of *N*=2,946 cells in this dataset. The second dataset was obtained from the GEO database under accession number GSE90975 and contains the gene expressions from single-cell analysis of neurodegeneration in microglia cells of mice [15]. We considered all *N*=944 cells and analyzed the *G*=101 differentially expressed genes from Figure S1 of [15]. This second real dataset is referred to as mouse microglia cell (MMC).

### Simulation studies

Using the simulated data, we fit our proposed model (HBFM) by running the Markov chain Monte Carlo (MCMC) sampling algorithm described in the “Methods” section. The stochastic EM approach was run for 2,000 iterations, after an initial warm-up period of 100 iterations, and samples from the last 200 iterations of this approach were used to obtain starting parameter values for the MCMC sampler. We ran the MCMC sampler for 4,000 iterations and used the last 1,000 iterations for inference.

Nine runs of HBFM were considered by selecting nine different choices for the number of factors: *F*= 5, 8, 10, 12, 15, 18, 20, 22, and 25. For each choice of *F*, we ran eight separate MCMC sampling chains in R [17], and used only the samples from the five chains with the highest average marginal likelihood for inference. The Deviance Information Criterion (DIC) was calculated using half the posterior variance of the deviance to estimate the effective number of parameters [18], and the number of factors *F* with the lowest DIC was selected as the “best” model choice. In the cases where *F*=25 was chosen as the “best” model, we ran an additional model with *F*=28 factors to ensure that the upper bound of our considered set was also the optimal choice for the number of factors. For each pair of genes *g* and *g*^′^ in the “best” model, we tested for a significant relationship by using a 95% credible interval (CI) for $\phantom {\dot {i}\!}\rho _{gg'}$.

To evaluate the performance of our model against other gene network methods, we ran the single-cell co-expression model LEAP [19] and the single-cell regulatory network models of PIDC [20] and SCODE [21] on the simulated data. After creating a symmetric correlation matrix with the LEAP package in R (i.e., selecting the maximum absolute correlation for each gene-gene pair), a permutation analysis was then performed with this package using a false discovery rate (FDR) of 5% to determine a cutoff for significant correlation values. PIDC was implemented in Julia [22] using the basic usage code available at https://github.com/Tchanders/NetworkInference.jl. For SCODE, we ran the R code available at https://github.com/hmatsu1226/SCODE and averaged the results of 50 separate trials using the same parameters as the example code provided on the GitHub page. The methods of LEAP and SCODE utilize a pseudotime estimation of the cells and the R package monocle [23] was used for this estimation.

We also included three popular network methods originally developed for bulk data in our simulation studies: partial correlation, Bayesian networks, and GENIE3 [24]. Partial correlation (PCORR) was implemented with the R package ppcor [25] using the Spearman partial correlation coefficient. We performed the Benjamini-Hochberg [26] procedure to control for FDR and defined 5% as the threshold for significant correlation values. Bayesian networks (BN) were constructed in R with the bnlearn package [27]. After learning a set of 1,000 bootstrap replicates with the hill-climbing algorithm, the optimal network was created using model averaging [27]. The analysis for GENIE3 was performed in R with the GENIE3 package using default parameters.

The methods of PIDC, SCODE, and GENIE3 output a matrix of scores/weights to quantify evidence towards each gene-gene regulatory link, but these methods do not determine a cutoff score/weight for identifying significant associations. To facilitate comparison across the networks from each method, we chose the threshold for PIDC, SCODE, and GENIE3 such that the number of edges in the constructed network was equal to the number of edges determined by our HBFM method. By matching the number of edges to our method, we provide a direct comparison between these methods and HBFM. In addition, SCODE and GENIE3 provide different scores/weights for the different directions of edges in the network; therefore, we followed the procedure by [8] and selected the directed edges with the higher magnitude to quantify the strengths of the gene-gene associations for these methods.

For each simulated dataset, we compared the significant gene-gene associations identified by each method to the “true” gene-gene associations created by the simulated network structure. The measures of true positive rate (TPR), FDR, area under the receiver operating characteristic curve (AUC), and number of significant edges in the estimated network were used to compare methods. When calculating the AUC, the inverse of the adjusted *p*-value (inverse of the approximate “*p*-value” in HBFM) for each gene-gene association was utilized for PCORR and HBFM, and for the other methods, the association value (or absolute value) provided for each network edge was used. We note that a different threshold for edge selection in PIDC, SCODE, and GENIE3 may impact the TPR and FDR results since the number of edges in the constructed network will change; however, the AUC results will remain unchanged by the threshold choice. We found that the FDRs for SCODE and GENIE3 tend to remain fairly stable across different threshold choices, and the FDR of PIDC tends to increase as the threshold increases. The performances of the different network methods are summarized in Tables 1 and 2.

**Table 1:** Results from simulation studies using data generated from the proposed methodology. The value of F for HBFM represents the number of factors in the “best” model choice, as determined by DIC

	**Sim 1: N=125,*F***_***sim***_**=10**					**Sim 2: N=125,*F***_***sim***_**=15**			
	**TPR**	**FDR**	**AUC**	**Edges**		**TPR**	**FDR**	**AUC**	**Edges**
**HBFM, F = 15**	0.760	0.153	0.927	314	**HBFM, F = 15**	0.640	0.111	0.820	306
**LEAP**	0.386	0.378	0.705	217	**LEAP**	0.341	0.275	0.665	200
**PIDC**	0.634	0.293	0.821	314^*^	**PIDC**	0.506	0.297	0.742	306^*^
**SCODE**	0.229	0.745	0.550	314^*^	**SCODE**	0.249	0.654	0.504	306^*^
**BN**	0.206	0.077	0.682	78	**BN**	0.186	0.037	0.672	82
**GENIE3**	0.540	0.398	0.746	314^*^	**GENIE3**	0.468	0.350	0.711	306^*^
**PCORR**	0.123	0.566	0.599	99	**PCORR**	0.148	0.442	0.602	113
	**Sim 3: N=500,*F***_***sim***_**=10**					**Sim 4: N=500,*F***_***sim***_**=15**			
	**TPR**	**FDR**	**AUC**	**Edges**		**TPR**	**FDR**	**AUC**	**Edges**
**HBFM, F = 25**	0.889	0.034	0.984	322	**HBFM, F = 25**	0.704	0.029	0.929	308
**LEAP**	0.743	0.608	0.741	664	**LEAP**	0.402	0.305	0.696	246
**PIDC**	0.794	0.137	0.915	322^*^	**PIDC**	0.621	0.143	0.866	308^*^
**SCODE**	0.249	0.730	0.501	322^*^	**SCODE**	0.216	0.701	0.578	308^*^
**BN**	0.277	0.040	0.751	101	**BN**	0.212	0.032	0.716	93
**GENIE3**	0.554	0.398	0.754	322^*^	**GENIE3**	0.466	0.357	0.729	308^*^
**PCORR**	0.300	0.266	0.683	143	**PCORR**	0.261	0.327	0.624	165
	**Sim 5: N=1000,*F***_***sim***_**=10**					**Sim 6: N=1000,*F***_***sim***_**=15**			
	**TPR**	**FDR**	**AUC**	**Edges**		**TPR**	**FDR**	**AUC**	**Edges**
**HBFM, F = 20**	0.909	0.076	0.973	344	**HBFM, F = 25**	0.624	0.070	0.904	285
**LEAP**	0.780	0.550	0.804	606	**LEAP**	0.591	0.541	0.680	547
**PIDC**	0.857	0.128	0.954	344^*^	**PIDC**	0.633	0.056	0.889	285^*^
**SCODE**	0.269	0.727	0.496	344^*^	**SCODE**	0.221	0.670	0.510	285^*^
**BN**	0.323	0.050	0.793	119	**BN**	0.247	0.037	0.710	109
**GENIE3**	0.603	0.387	0.764	344^*^	**GENIE3**	0.440	0.344	0.700	285^*^
**PCORR**	0.403	0.291	0.720	199	**PCORR**	0.294	0.251	0.669	167

^*^Number of edges fixed to match HBFM

**Table 2:** Results from simulation studies using data generated from the NORTA algorithm. The value of F for HBFM represents the number of factors in the “best” model choice, as determined by DIC

	**Sim 7: N=125, Edges**_**Sim**_**=350**					**Sim 8: N=125, Edges**_**Sim**_**=425**			
	**TPR**	**FDR**	**AUC**	**Edges**		**TPR**	**FDR**	**AUC**	**Edges**
**HBFM, F = 18**	0.817	0.043	0.927	299	**HBFM, F = 18**	0.638	0.000	0.942	271
**LEAP**	0.700	0.377	0.819	393	**LEAP**	0.466	0.423	0.630	343
**PIDC**	0.714	0.164	0.875	299^*^	**PIDC**	0.560	0.122	0.817	271^*^
**SCODE**	0.243	0.716	0.491	299^*^	**SCODE**	0.169	0.734	0.540	271^*^
**BN**	0.211	0.026	0.718	76	**BN**	0.148	0.000	0.689	63
**GENIE3**	0.349	0.592	0.576	299^*^	**GENIE3**	0.306	0.520	0.558	271^*^
**PCORR**	0.157	0.396	0.559	91	**PCORR**	0.132	0.434	0.564	99
	**Sim 9: N=500, Edges**_**Sim**_**=350**					**Sim 10: N=500, Edges**_**Sim**_**=425**			
	**TPR**	**FDR**	**AUC**	**Edges**		**TPR**	**FDR**	**AUC**	**Edges**
**HBFM, F = 22**	0.871	0.041	0.993	318	**HBFM, F = 20**	0.727	0.019	0.947	315
**LEAP**	0.880	0.548	0.909	681	**LEAP**	0.744	0.557	0.741	713
**PIDC**	0.857	0.057	0.941	318^*^	**PIDC**	0.722	0.025	0.933	315^*^
**SCODE**	0.249	0.726	0.571	318^*^	**SCODE**	0.240	0.676	0.520	315^*^
**BN**	0.251	0.064	0.764	94	**BN**	0.195	0.057	0.669	88
**GENIE3**	0.366	0.597	0.601	318^*^	**GENIE3**	0.355	0.521	0.569	315^*^
**PCORR**	0.220	0.280	0.609	107	**PCORR**	0.198	0.408	0.571	142
	**Sim 11: N=1000, Edges**_**Sim**_**=350**					**Sim 12: N=1000, Edges**_**Sim**_**=425**			
	**TPR**	**FDR**	**AUC**	**Edges**		**TPR**	**FDR**	**AUC**	**Edges**
**HBFM, F = 25**	0.966	0.048	0.989	355	**HBFM, F = 25**	0.831	0.033	0.968	365
**LEAP**	0.926	0.623	0.870	859	**LEAP**	0.849	0.529	0.809	767
**PIDC**	0.957	0.056	0.977	355^*^	**PIDC**	0.807	0.060	0.940	365^*^
**SCODE**	0.303	0.701	0.506	355^*^	**SCODE**	0.191	0.778	0.625	365^*^
**BN**	0.343	0.084	0.756	131	**BN**	0.233	0.075	0.734	107
**GENIE3**	0.414	0.592	0.596	355^*^	**GENIE3**	0.374	0.564	0.565	365^*^
**PCORR**	0.297	0.373	0.654	166	**PCORR**	0.228	0.276	0.612	134

^*^Number of edges fixed to match HBFM

From the simulation results, we see that our methodology performs quite well across the different scenarios, as HBFM has consistently high power and low FDRs. In Fig. 1, we visually provide comparisons of the correlation structures estimated by HBFM to the “true” correlation structures of Sim 3 and Sim 4 to illustrate that our method is able to recover the underlying correlation structures. The magnitude and direction of the estimated correlation structures produced by HBFM tend to resemble the magnitude and direction of the “true” correlation structures.

When examining the performances of all methods in the simulation studies, our model outperforms the other methods across the TPR and AUC performance measures. Even when the data was generated via the NORTA algorithm (Sim 7 - 12), our HBFM method performs better than the other considered methods. LEAP tends to identify larger numbers of edges than the other methods, which leads to higher TPR than HBFM in some simulations. However, HBFM has a higher AUC and much lower FDR than LEAP in every considered simulated dataset. In Sim 6, HBFM and PIDC perform very comparably when the number of edges is the same. While PIDC has a slightly higher TPR and lower FDR at this threshold, HBFM does have the higher AUC. The FDR of our method is also reasonably controlled to a nominal level, especially compared to the FDRs of LEAP, SCODE, GENIE3, and PCORR. While BN had lower FDRs than HBFM in some of the simulations, it also identified the fewest number of edges and had lower TPR and AUC than HBFM. Example heatmaps of the networks produced by all considered methods are provided in Additional file 1 (Figures S1 - S3).

When using DIC as the criterion for our model selection, the best-fitting model often contains more factors than the “true” simulated structure in the examples we’ve considered so far. However, we note that the additional factors provide more opportunities to explore different factor structures within the model during MCMC sampling. For example, a single factor from a model with *F*=10 may be split into several factors when using a model with *F*=20. Therefore, it is not surprising that the “best” model choices contain more factors than the “true” number of factors, *F*_*sim*_, as these models are more likely to explore the high regions of the posterior because they are less likely to get stuck during sampling.

### Case studies

The same network methods described in the “Simulation studies” section were applied to the two real datasets. Since the “true” network structure of the real data is unknown, we constructed three reference protein-protein interaction networks with the STRING database [28] for each dataset to compare across the different methods. These reference networks were created by adjusting the threshold for the minimum required interaction score between pairs of proteins: high confidence (minimum score of 0.700), medium confidence (minimum score of 0.400), and low confidence (minimum score of 0.150). STRING computes these scores by combining the probabilities of different evidence sources (e.g., text mining, experiments, databases) and correcting for the probability of observing the interactions by random chance [29]. This is, of course, an imperfect reference as any method may detect novel interactions that have not been previously published. Likewise, some entries in STRING may represent published false positives. However, on average, the method producing the network most similar to the known and predicted protein-protein interaction STRING reference set should be considered as the network most consistent with biological literature.

Because the methods of PIDC, SCODE, and GENIE3 do not have default parameters to determine a cutoff score/weight for identifying significant associations, we have selected the same number of top edges from each considered method and used those top edges to evaluate the performance of the methods in the real data analysis. For each method, we constructed a network and obtained the top 322 most significant gene-gene pairs, out of the 1,128 possible pairs, for comparison in the MBSC analysis and the top 1,600 most significant pairs, out of the 5,050 possible pairs, for comparison in the MMC analysis. These values represent the number of protein-protein interactions in the low confidence STRING reference sets. From the nine different numbers of factors considered for HBFM, we selected *F*=25 factors as the “best” choice for both the MBSC and MMC data because this factor choice had the lowest DIC.

**Fig. 2:**
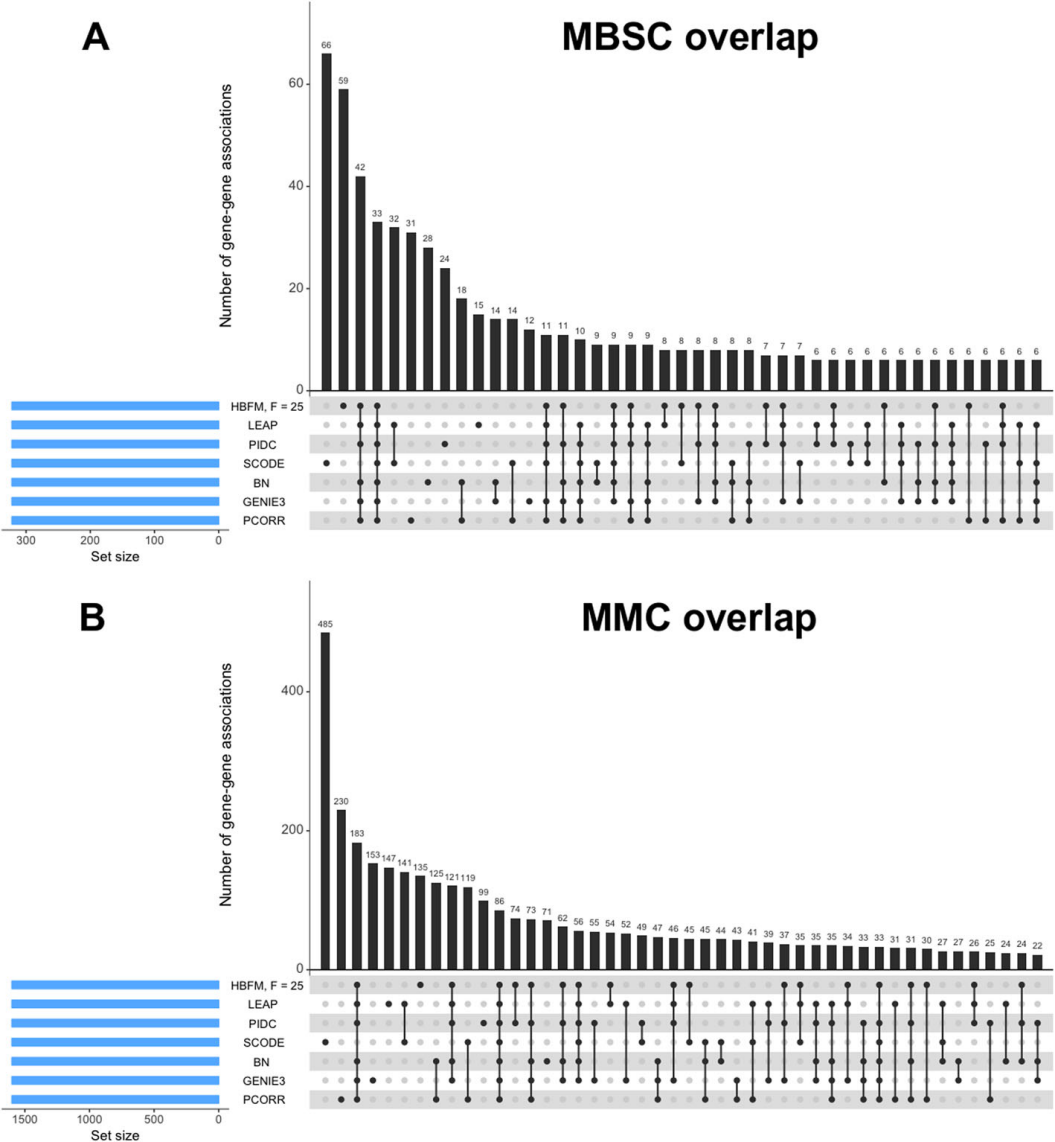
**a** UpSet plot of the top 322 gene-gene associations as determined by seven different methods for the MBSC dataset. **b** UpSet plot of the top 1,600 gene-gene associations as determined by seven different methods for the MMC dataset

The UpSet plots [30] for the intersection between the top 322 associations in the MBSC dataset and the top 1,600 associations in the MMC dataset identified by each network method is displayed in Fig. 2. The dark circles in each column of the UpSet plot indicate the methods associated with the intersection and the bar above each column represents the number of gene-gene pairs in the intersection. Interestingly, only 33 and 86 associations were common among all seven methods in the MBSC and MMC datasets, respectively.

Table 3 displays the comparisons of the top associations from each method to the reference networks. In the MBSC analysis, HBFM has the most associations in common with each STRING reference network. The 19 high confidence STRING interactions identified by HBFM form a network of 14 distinct genes: *Penk*, *Calb2*, *Reln*, *Npy*, *Sst*, *Lhx6*, *Pvalb*, *Crh*, *Vip*, *Tbr1*, *Foxp2*, *Calb1*, *Cck*, and *Pax6*. According to the STRING database, these genes are associated with 127 significantly enriched biological process gene ontology (GO) terms that include behavior, cerebral cortex development, learning or memory, and forebrain development. LEAP also matched the same number of high confidence interactions as our method in the MBSC analysis but did not match as many of the medium and low confidence interactions.

**Table 3:** The overlap between the top 322 gene-gene associations in the MBSC dataset and the top 1,600 gene-gene associations in the MMC dataset for each network method. Reference networks were created by the STRING database

	**MBSC reference set**			**MMC reference set**		
	**High**	**Medium**	**Low**	**High**	**Medium**	**Low**
**HBFM, F=25**	19	50	113	618	707	926
**LEAP**	19	45	100	263	357	678
**PIDC**	13	40	95	460	559	838
**SCODE**	9	27	74	167	247	517
**BN**	12	40	96	384	474	783
**GENIE3**	12	38	94	338	434	733
**PCORR**	14	40	102	263	346	582
**Reference total**	42	116	322	697	897	1600

For the MMC dataset, HBFM again has the highest number of associations in common with each STRING reference network. When comparing the methods to the high confidence STRING network, HBFM matched 618 out of the 697 (88.6%) interactions while PIDC had the second highest overlap matching only 460 of the 697 (66.0%) interactions. The network of 618 high confidence interactions identified by HBFM consists of 78 distinct genes that are associated with 271 significantly enriched biological process GO terms. The most significant GO terms for these genes include translation, peptide metabolic process, and organonitrogen compound biosynthetic process. Lists of the high confidence interaction genes detected by our method in both the MBSC and MMC analyses and their associated significantly enriched biological process GO terms are provided in Additional file 2.

As an additional evaluation of our HBFM model, we created 100 posterior predictive datasets (PPDs) [18] from each chain of the MMC analysis (500 PPDs in total) and compared the overdispersion and proportion of zeros in these datasets to the overdispersion and proportion of zeros in the MMC dataset. Each count *Y*_*gi*_ of the PPDs was generated from Poisson(*μ*_*gi*_), with *μ*_*gi*_ modeled from Eq. (1) using parameter estimates (with the exception of the ***λ***_***i***_ parameters) from different iterations of the MCMC sampler. The ***λ***_***i***_ values were drawn randomly from Lognormal (0,*ϕ*_*f*_).

**Fig. 3:**
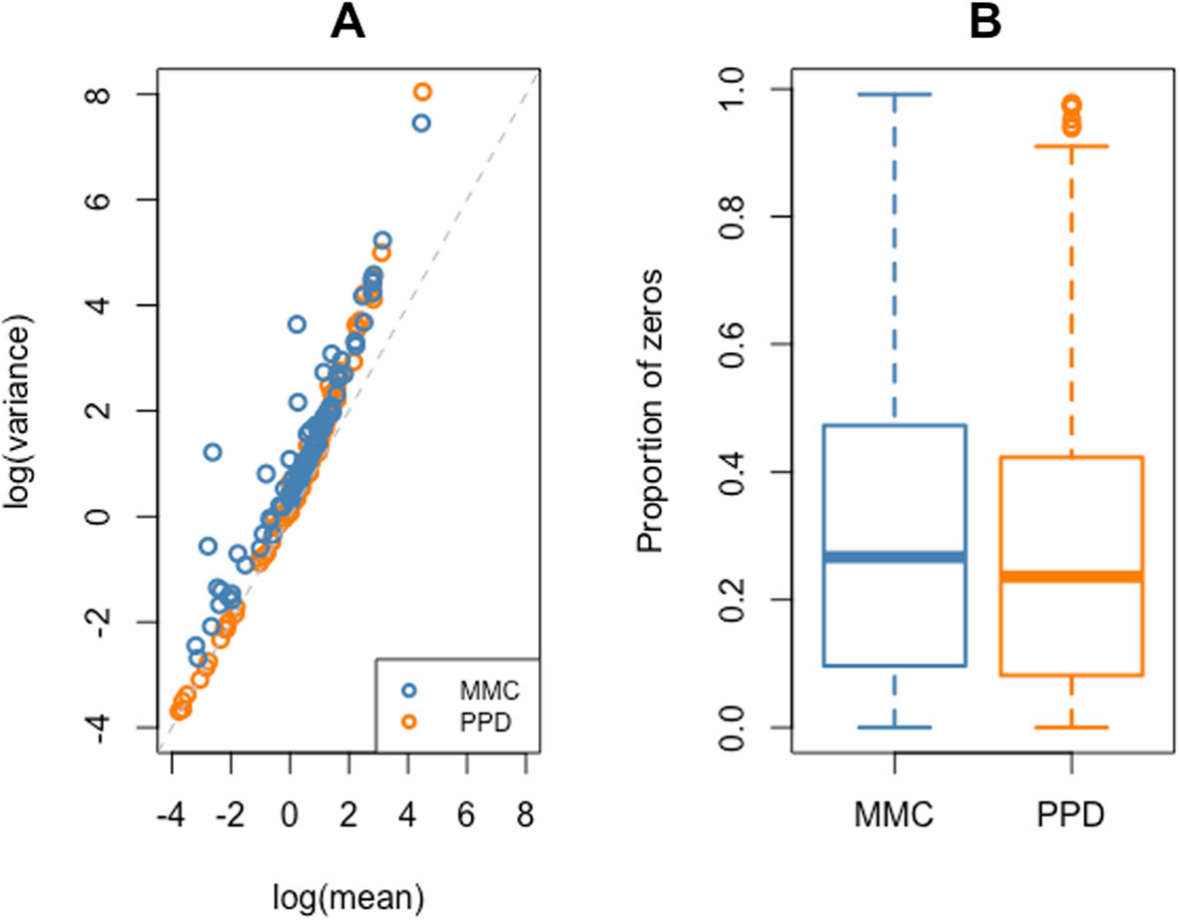
Example comparison between the MMC dataset and one representative PPD generated by HBFM. **a** The log(variance) vs. log(mean) scatterplot for each gene. **b** Boxplots of gene-specific proportion of zeros

In Fig. 3a, the log(variance) is plotted against the log(mean) across all *G*=101 genes for the real expressions in the MMC dataset and the estimated expressions from a single representative PPD. Both datasets display high cell-to-cell variability, as expected of scRNA-seq data. In fact, even with the choice of Poisson for the (conditional) distribution of the counts, the PPDs generated from the parameters estimated from the MMC dataset tend to generate variability that is comparable to the variability observed in the real data. We can see that many genes from the PPD are overdispersed, especially those with log(means) greater than 1, as in the true MMC data. From Fig. 3b, the gene expression in the MMC data is zero-inflated as the proportion of zero values for each gene ranged between 0 and 0.99. In the PPD, the proportion of zeros for each gene tended to be only slightly lower than what was observed in the real dataset. Nevertheless, the proportion of zero expressions were still quite high and variable across the genes in the PPD.

**Fig. 4:**
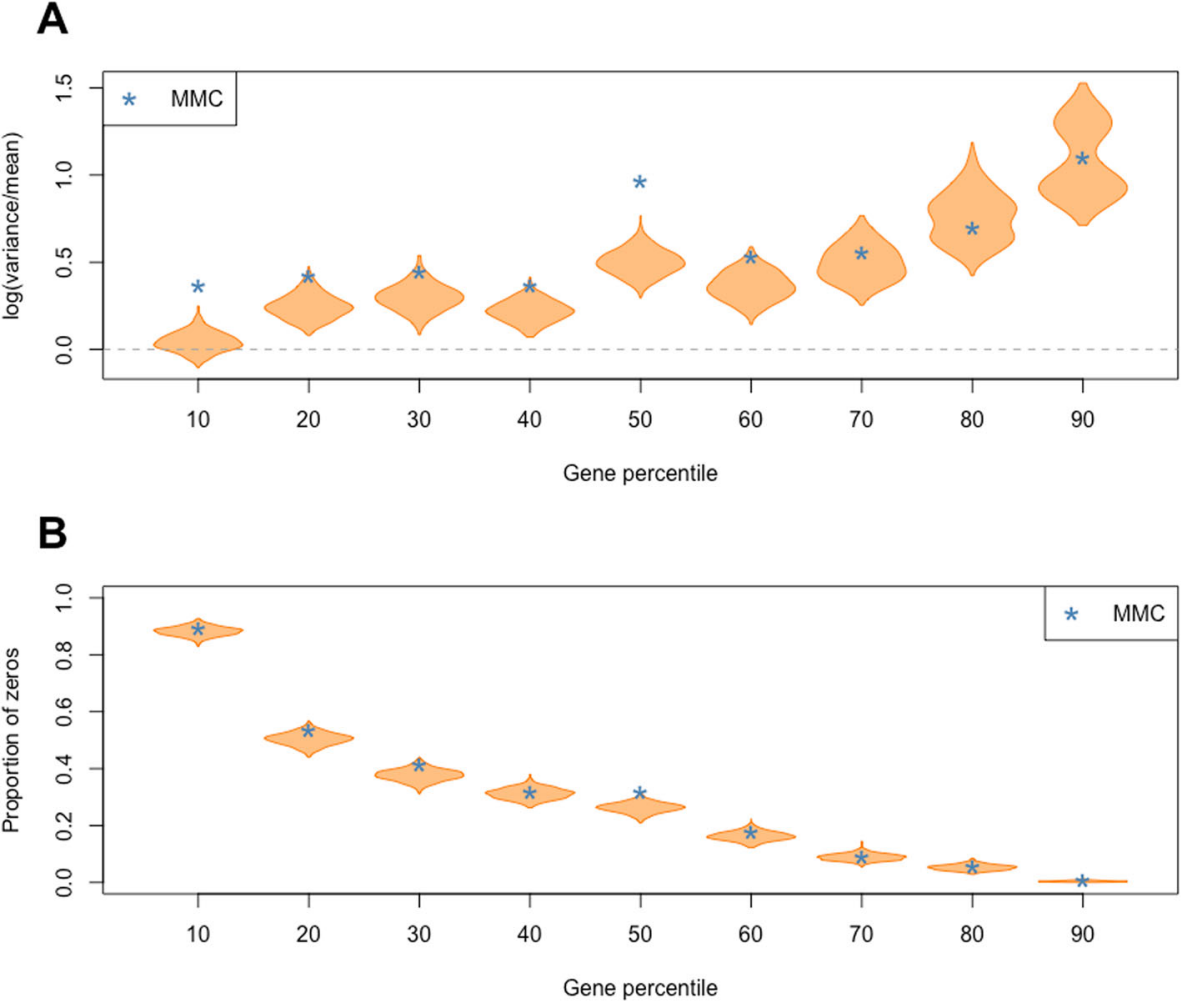
Properties of PPD estimates from a sample of nine genes in the MMC dataset. Genes were selected based on percentiles (10th through 90th) of average gene expression. **a** Violin plots of estimated log(variance/mean) for each gene across all PPDs. **b** Violin plots of estimated gene-specific proportion of zeros across all PPDs. The blue stars represent the true values from the MMC dataset

To further analyze the PPDs generated by HBFM, we selected nine genes from the MMC dataset that represent the 10th through 90th percentiles of average gene expression and examined the log(variance/mean) and proportion of zeros of these genes across all PPDs. Figure 4a illustrates that across the PPDs, the estimated log(variance/mean) for most of the genes is greater than 0, indicating variances that are larger than their corresponding means. Also, for a majority of these genes, the true log(variance/mean) value is captured across the PPD estimates. The estimated proportion of zeros for these genes across the PPDs also capture the true proportion of zeros from the MMC dataset, as displayed in Fig. 4b.

## Discussion

In this manuscript, we have presented a hierarchical Bayesian factor model (which we have referred to as HBFM) for constructing GCNs from scRNA-seq data. We do note that our methodology constructs undirected networks to identify gene-gene associations unlike some of the other considered methods (BN, GENIE3, LEAP, and SCODE) that do provide directed edges to infer causal relationships. Inference between undirected and directed graphs may not be fully comparable, but rather than limit our comparison to only methods for undirected graphs, we have included methods that estimate directed graphs and have adjusted the comparisons using common strategies from the literature.

The number of genes (*G*) in the simulated and real datasets presented in this manuscript is smaller than what is often considered for other scRNA-seq data problems, such as clustering cells/genes and detection of differentially expressed genes. However, the use of a smaller pre-screened set of genes is common among other complex network methods [5, 31]. In part, this is due to the GCN being determined by *G*∗(*G*−1)/2 correlations, a quadratic number of parameters, making it difficult to numerically and graphically communicate results for large *G*. While constructing a GCN as an exploratory analysis from an entire dataset is possible with our method, it may not be computationally practical. HBFM performs Bayesian inference via iterative MCMC, which can become computationally expensive as the number of genes (*G*) and number of cells (*N*) increase.

In light of these computational considerations, we typically recommend the user consider some initial analysis such as clustering or differential expression to determine a smaller set of genes, generally 100 or fewer, before using HBFM to estimate the GCN. On a system with an Intel Core i7 processor (3.5 GHz) and 8 GB of RAM, the average running time for a single chain of HBFM with *F*=25 factors was 20.1 hours for the MBSC data (*G*=48,*N*=2,946) and 11.2 hours for the MMC data (*G*=101,*N*=944). Also, when it comes to choosing an appropriate number of factors (*F*) for our methodology, we found that the correlation structure estimated by HBFM was reasonably stable across *F* values greater than 10 in the simulation studies (see Figure S4 in Additional file 1). For the analyses in this manuscript, we considered nine potential *F* values and ran eight chains for each choice of *F* in parallel so that we could produce a thorough investigation of the performance of our proposed methodology. However, a smaller set of *F* values (with all *F* values greater than 10) can be considered for practical applications of HBFM.

In our methodology, the distribution of count values is defined to follow a Poisson distribution, conditional on the latent factors ***λ***_***i***_. While we acknowledge that the negative binomial distribution tends to be the preferred choice for modeling overdispersed data, the latent factors of HBFM are random effects that help account for the additional variability across samples. After marginalizing out ***λ***_***i***_, *E*(*Y*_*gi*_)=*β*_*g*_ and $Var\left (Y_{gi}\right)=\beta _{g} + \beta _{g}^{2} \left (exp\left \{-\phi _{f}\lvert \alpha _{gf} \rvert \right \}-1\right)$. As illustrated in the PPDs generated from the real MMC data, HBFM is able to generate overdispersed and zero-inflated data that is consistent with the features of the real data. Hence, the use of a Poisson distribution is not a meaningful drawback. As a potential extension of our methodology, a new parameter *ε*_*gi*_∼*Gamma*(*r*_*g*_,*r*_*g*_) could be added to our model such that *Y*_*gi*_∼*Poisson*(*μ*_*gi*_*ε*_*gi*_) with *μ*_*gi*_ defined from Eq. (1). The conditional distribution of *Y*_*gi*_ would be Poisson but marginally the distribution would be negative binomial with mean *μ*_*gi*_ and dispersion parameter $d_{g}=\frac {1}{r_{g}}$. Our preliminary analyses examining this conditionally negative binomial model version indicated no improvement in inference.

We also note that the high resolution of scRNA-seq technology allows researchers the opportunity to estimate “pseudotime” and obtain a temporal ordering of cells [23, 32]. The general idea is that at any given time, a cell population will consist of cells that are at different stages of differentiation and development, and cells in different stages will express different sets of genes. Our method does not directly take pseudotime into account, but the latent factors (***λ***) are likely to adapt and capture this contribution on the gene expression.

## Conclusion

The results from our simulation studies demonstrate that HBFM is able to identify true co-expressions while maintaining a nominal FDR across different numbers of cells and different network structures, even when the data was simulated from a structure that differs from our proposed methodology. Our case study analyses with the MBSC and MMC datasets also demonstrate the practical use of HBFM for determining significant gene-gene associations, as our model was able to detect more known and predicted protein-protein interactions from the STRING database than the competitor network methods. Overall, our proposed hierarchical Bayesian factor model is a promising method for discovering gene-gene associations in future scRNA-seq network analyses.

## Methods

### Hierarchical Bayesian factor model

Let *Y*_*gi*_ be the (count) expression for gene *g* (*g*=1,…,*G*) in cell *i* (*i*=1,…,*N*). We assume each expression comes from the Poisson(*μ*_*gi*_) distribution, where the mean *μ*_*gi*_ is modeled through the representation

1 $$\begin{array}{@{}rcl@{}}  \mu_{gi} = \beta_{g} \prod\limits_{f=1}^{F} exp\left\{-\frac{\phi_{f}}{2}\lvert \alpha_{gf} \rvert \right\}\lambda_{if}^{~\alpha_{gf}}. \end{array} $$

Here, the parameter *β*_*g*_ denotes the average expression for gene *g*. For each cell *i*, there are *F* associated factors $\boldsymbol {\lambda _{i}}=\left \{\lambda _{i1},\dots,\lambda _{iF}\right \}$ that impact the expression. These factors are strictly positive and come from a Lognormal (0,*ϕ*_*f*_) distribution. We can think of each factor as representing a distinct attribute (e.g., cell stage, pseudotime point) that will only influence a specific set of related gene expressions. The exponent of the *f*th factor *λ*_*if*_ is *α*_*gf*_∈{−1,0,1}, and by using this set of discrete exponents for the factors, the expression for gene *g* is impacted only by the factors with *α*_*gf*_=−1 or 1. The adjustment term of $exp\left \{-\frac {\phi _{f}}{2}\lvert \alpha _{gf} \rvert \right \}$ is included in Eq. (1) to ensure that *E*(*Y*_*gi*_) is equal to *β*_*g*_ (after marginalizing out ***λ***_***i***_) regardless of the *α*_*gf*_ values.

Our defined factor structure provides the flexibility required to account for the typical cell-to-cell variability of scRNA-seq data. For a given *f*, *λ*_*if*_ is unique to each cell and is only activated for a particular gene when *α*_*gf*_≠0. If the activated factors $\lambda _{if}^{~\alpha _{gf}}$ for a given gene are much smaller than 1 (near zero), then *μ*_*gi*_ will be very small and account for the high proportion of zeros typical of this data. Conversely, very large values of the factors will increase *μ*_*gi*_ (relative to the baseline *β*_*g*_) and accommodate the occasional extremely large count. We note here that *Y*_*gi*_ follows a Poisson distribution conditional on the ***λ***_***i***_ terms. However, the variance of *Y*_*gi*_, marginal on ***λ***_***i***_, is equal to $\beta _{g} + \beta _{g}^{2} \left (exp\left \{-\phi _{f}\lvert \alpha _{gf} \rvert \right \}-1\right)$. Thus, *Y*_*gi*_ is conditionally Poisson but marginally overdispersed. So, despite the choice of Poisson for the distribution of the count, our model is able to capture the high proportion of zeros and large variance typical of single-cell data.

To finish specification of our Bayesian model, prior distributions for the remaining parameters must be defined. We use a conditionally conjugate, non-informative prior for the average expression of gene *g*, *β*_*g*_∼*Gamma*(0.001,0.001). The prior for the scale parameter of the factors is *ϕ*_*f*_∼*Lognormal*(*h*_1_,*h*_2_), where *h*_1_∼*Normal*(0,100) and *h*_2_∼*Inverse* *Gamma*(1,1). For the exponent parameters, the prior is |*α*_*gf*_|∼*Bernoulli*(*θ*_*f*_) with *θ*_*f*_∼*Beta*(1,1). Here, we define $P\left (\alpha _{gf} = 1\right) = P\left (\alpha _{gf} = -1\right) = \frac {\theta _{f}}{2}$. Consequently, *P*(*α*_*gf*_=0)=1−*θ*_*f*_. The number of associated factors *F* is often unknown, but one can fit multiple models with different numbers of factors and choose the most suitable model based on a comparison of a model selection statistic such as the DIC described in [18].

### Network structure

Posterior samples for model parameters are obtained with the MCMC algorithm defined later in the “Model inference” section. At each iteration of the MCMC, a correlation matrix is computed based on the current set of parameters, and we infer a GCN by examining the posterior distribution of this correlation matrix. Under our proposed model, the sparse ***α***={*α*_*gf*_}_(*g*,*f*)_ matrix imposes a crude network structure on the gene expressions. Consider two genes *g* and *g*^′^, where *g*≠*g*^′^. If $\phantom {\dot {i}\!}\alpha _{gf}\alpha _{g'f} \neq 0$ for some *f*, the expressions *Y*_*gi*_ and $\phantom {\dot {i}\!}Y_{g'i}$ are both impacted by the shared factor *λ*_*if*_. Conversely, if genes *g* and *g*^′^ have no shared factors ($\phantom {\dot {i}\!}\alpha _{gf}\alpha _{g'f}=0$ for all *f*), these genes are conditionally independent. To quantify the association between gene *g* and gene *g*^′^, we examine the correlation (after marginalizing out ***λ***_***i***_) between the values of *log*(*μ*_*gi*_) and $\phantom {\dot {i}\!}log\left (\mu _{g'i}\right)$.

We motivate our decision to use this specific correlation structure by considering the matrix $\boldsymbol {\tilde {A}} = \boldsymbol {\alpha \alpha ^{T}}$. The (*g*,*g*^′^) element of this *G*×*G* matrix provides a summation of the associated factors that are active in both genes *g* and *g*^′^ since $\tilde {a}_{g,g'} = {\sum \nolimits }_{f=1}^{F} \alpha _{gf}\alpha _{g'f}\phantom {\dot {i}\!}$. When $\tilde {a}_{g,g'} > 0$, the two genes have more factors with the same association (i.e., $\phantom {\dot {i}\!}\alpha _{gf}=\alpha _{g'f}=1$ or $\phantom {\dot {i}\!}\alpha _{gf}=\alpha _{g'f}=-1$) than factors with opposite associations (i.e., *α*_*gf*_=1 and $\phantom {\dot {i}\!}\alpha _{g'f}=-1$ or vice versa). Conversely, when $\tilde {a}_{g,g'} < 0$, the genes have more factors with opposite associations than factors with the same association. If $\tilde {a}_{g,g'} = 0$, then either no factors are in common between the genes or the number of factors with the same association is equal to the number of factors with opposite associations for those genes.

By recognizing that factors with a larger variance *ϕ*_*f*_ will have a greater influence on the joint expression, we can weigh the shared factors by their variance. In fact, this weighted expression is exactly equal to the covariance (marginally over ***λ***_***i***_) between *log*(*μ*_*gi*_) and $\phantom {\dot {i}\!}log\left (\mu _{g'i}\right)$,

$$\begin{array}{@{}rcl@{}} Cov \left[log\left(\mu_{gi}\right),log\left(\mu_{g'i}\right)\right] = \sum\limits_{f=1}^{F} \phi_{f}\alpha_{gf}\alpha_{g'f}~. \end{array} $$

The active factors also increase the variance for *log*(*μ*_*gi*_),

$$\begin{array}{@{}rcl@{}} Var\left[log\left(\mu_{gi}\right)\right] = \sum\limits_{f=1}^{F} \phi_{f}\alpha_{gf}^{2} ~, \end{array} $$

which is important when addressing the zeros and overdispersion of scRNA-seq data. From these covariance and variance expressions, the correlation between *log*(*μ*_*gi*_) and $\phantom {\dot {i}\!}log(\mu _{g'i})$ is defined as

2 $$\begin{array}{@{}rcl@{}}  Corr\left[log\left(\mu_{gi}\right),log\left(\mu_{g'i}\right)\right] = \rho_{gg'}= \frac{{\sum\nolimits}_{f=1}^{F} \phi_{f}\alpha_{gf}\alpha_{g'f}} {\sqrt{\left({\sum\nolimits}_{f=1}^{F} \phi_{f}\alpha_{gf}^{2}\right) \left({\sum\nolimits}_{f=1}^{F} \phi_{f}\alpha_{g'f}^{2}\right)}}. \end{array} $$

We illustrate the mechanics of this correlation structure by considering just one factor *f*. If gene *g* and gene *g*^′^ have the same association with this given factor, the correlation between *log*(*μ*_*gi*_) and $\phantom {\dot {i}\!}log\left (\mu _{g'i}\right)$ is 1. When gene *g* has a positive association with factor *f* and gene *g*^′^ has a negative association with factor *f*, the correlation is −1. Additionally, if factor *f* is inactive for either of the genes, the correlation is 0. The significance of each correlation is determined by analyzing the credible interval (CI) of $\rho _{gg'}\phantom {\dot {i}\!}$ in the posterior distribution, as described in the “Network inference” section.

We note that each gene must have at least one active factor for our correlation structure in Eq. (2) to be defined since *Var*[*log*(*μ*_*gi*_)] is equal to 0 if all of the factors are inactive. Utilizing the correlation structure (after marginalizing out ***λ***_***i***_) between *Y*_*gi*_ and $\phantom {\dot {i}\!}Y_{g'i}$ would avoid this issue, but the additional *β*_*g*_ term in the variance leads to a correlation structure dependent on the average expression for each gene. For this reason, we do not focus on the correlation structure between *Y*_*gi*_ and $\phantom {\dot {i}\!}Y_{g'i}$. Throughout, if (2) is $\frac {0}{0}$, we define this correlation as zero to match the zero value for $\phantom {\dot {i}\!}Corr\left (Y_{gi}, Y_{g'i}\right)$.

### Model inference

The posterior distribution for our hierarchical Bayesian model is complex, and so MCMC is required for inference. For simplicity in our posterior distribution notations, let $ \psi _{gif} = \prod _{f'\ne f} exp\left \{-\frac {\phi _{f'}}{2} \left \lvert \alpha _{gf'} \right \rvert \right \} \lambda _{if'}^{~\alpha _{g}f'}. $ We utilize an MCMC sampler that iterates through the following steps:

1. For $g=1,\dots,G$, update $\beta _{g} \sim Gamma \left (0.001+{\sum \nolimits }_{i=1}^{N} y_{gi} ~,~ 0.001+{\sum \nolimits }_{i=1}^{N} \prod \nolimits _{f=1}^{F} exp\left \{-\frac {\phi _{f}}{2} \lvert \alpha _{gf} \rvert \right \} \lambda _{if}^{~\alpha _{g}f}~ \right)$.
2. For $f=1,\dots,F$, update $\theta _{f} \sim Beta \left (1 + {\sum \nolimits }_{g=1}^{G} \lvert \alpha _{gf} \rvert ~,~ 1 + G - {\sum \nolimits }_{g=1}^{G} \lvert \alpha _{gf} \rvert \right)$.
3. For all *g*,*f*, sample *α*_*gf*_ from a multinomial distribution with
  $p\left (\alpha _{gf} = 0 | \cdots \right) = \frac {A}{A+B+C}$,
  $p\left (\alpha _{gf} = 1 | \cdots \right) = \frac {B}{A+B+C}$,
  $p\left (\alpha _{gf} = -1 | \cdots \right) = \frac {C}{A+B+C}$.
  Here, *A*,*B*, and *C* are defined as
  $A = \left (1-\theta _{f}\right) exp\left \{-\beta _{g} {\sum \nolimits }_{i=1}^{N} \psi _{gif} \right \}$,
  $B = \left (\frac {\theta _{f}}{2}\right)exp\left \{-\beta _{g} {\sum \nolimits }_{i=1}^{N} exp\left \{-\frac {\phi _{f}}{2} \right \} \lambda _{if} \psi _{gif} \right \}$,
  $C = \left (\frac {\theta _{f}}{2}\right) exp\left \{-\beta _{g} {\sum \nolimits }_{i=1}^{N} \frac {exp\left \{-\frac {\phi _{f}}{2}\right \}}{\lambda _{if}}\psi _{gif} \right \}$.
4. Update $h_{1} \sim Normal\left (\frac {1/h_{2}}{1/100+F/h_{2}}* {\sum \nolimits }_{f=1}^{F} log\left (\phi _{f}\right), \left (1/100 + F/h_{2}\right)^{-1}\right)$.
5. Update $h_{2} \sim Inverse~Gamma\left (\frac {F}{2}+1, \frac {{\sum \nolimits }_{f=1}^{F}\left (log\left (\phi _{f}\right)-h_{1}\right)^{2}}{2}+1\right)$.
6. For $f = 1,\dots,F$, use a Metropolis-Hastings step to update *ϕ*_*f*_. The posterior distribution for *ϕ*_*f*_ is
  $p\left (\phi _{f} | \cdots \right) \propto \phi _{f}^{~-\frac {N}{2}-1} exp\left \{ - \left (\frac {\phi _{f}}{2} {\sum \nolimits }_{g=1}^{G}{\sum \nolimits }_{i=1}^{N}\left \lvert \alpha _{gf} \right \rvert y_{gi} + \frac {{\sum \nolimits }_{i=1}^{N} log\left (\lambda _{if}\right)^{2}}{2\phi _{f}} +\right. \right.$
  $\left.\left.\qquad \frac {(log(\phi _{f})-h_{1})^{2}}{2h_{2}} + {\sum \nolimits }_{g=1}^{G} \beta _{g} exp\left \{-\frac {\phi _{f}}{2} \lvert \alpha _{gf} \rvert \right \} {\sum \nolimits }_{i=1}^{N} \lambda _{if}^{~\alpha _{g}f}~ \psi _{gif} \right) \right \}$.
  We propose a candidate value for $\phi _{f}^{(c)}$ through a pseudo-random walk from Lognormal (*ϕ*_*f*_,*σ*^2^) and accept this value with the usual Metropolis-Hastings ratio. If factor *f* is not active for any gene (i.e., ${\sum \nolimits }_{g=1}^{G} \lvert \alpha _{gf} \rvert = 0$), then update *ϕ*_*f*_ from the Lognormal (*h*_1_,*h*_2_) prior.
7. For all *i*,*f*, use a Metropolis-Hastings step to update *λ*_*if*_. By defining
  $\kappa = {\sum \nolimits }_{g=1}^{G} y_{gi} \alpha _{gf} $,
  $\tau = 2{\sum \nolimits }_{g=1}^{G} I\left (\alpha _{gf}=1\right)\beta _{g} ~exp\left \{-\frac {\phi _{f}}{2} \right \} \psi _{gif}$,
  $\chi = 2{\sum \nolimits }_{g=1}^{G} I\left (\alpha _{gf}=-1\right)\beta _{g} ~exp\left \{-\frac {\phi _{f}}{2} \right \} \psi _{gif}$, where *I*(·) represents an indicator variable, the posterior distribution for *λ*_*if*_ is
  $p\left (\lambda _{if} | \cdots \right) \propto \lambda _{if}^{~\kappa -1} exp\left \{ -\frac {1}{2} \left (\tau \lambda _{if} + \frac {\chi }{\lambda _{if}} + \frac {log\left (\lambda _{if}\right)^{2}}{\phi _{f}} \right)\right \}$.
  This posterior has a similar appearance to a generalized inverse Gaussian (GIG) distribution with an extra exponential term $\left (\frac {log\left (\lambda _{if}\right)^{2}}{\phi _{f}} \right)$. To that end, we propose a candidate value for $\lambda _{if}^{(c)}$ from GIG (*κ*,*bτ*,*bχ*), where the multiplicative factor of *b* on *τ* and *χ* is used to create thicker tails in the proposal distribution. For our sampling scheme, we set *b* to 0.9. Acceptance of the candidate value is determined by the typical Metropolis-Hastings rules. If *τ*=*χ*=0, factor *f* is not active and we update *λ*_*if*_ from the Lognormal (0,*ϕ*_*f*_) prior.

Due to the large number of model parameters and complexity of the posterior distribution, it is possible for the MCMC sampler to get stuck exploring a local mode of the posterior rather than exploring the entire posterior distribution. This is particularly an issue with the one-at-a-time sampling for ***α***, which does not allow for large scale moves such as splitting or combining factors. To address this sampling problem, we implement a stochastic EM approach [33, 34] to obtain initial values for our MCMC algorithm.

For the stochastic EM approach, we run the usual MCMC sampler but replace sampling with optimization in several of the steps. Specifically, we optimize the following steps of the sampler:

1. For $g=1,\dots,G$, update *β*_*g*_ to its conditional posterior mode.
2. For all *g*,*f*, select the value of *α*_*gf*_ with the highest probability: *p*(*α*_*gf*_=0|⋯), *p*(*α*_*gf*_=1|⋯), or *p*(*α*_*gf*_=−1|⋯).
3. For $f = 1,\dots,F$, find *ϕ*_*f*_ that optimizes its respective conditional posterior distribution. In this step, we utilize the *optimize* function from the base packages in R [17].

After randomly selecting starting values and running an initial MCMC sampling warm-up period, the stochastic EM approach is implemented for a number of iterations (e.g., 2,000 iterations) to ensure stabilization. Parameter estimates are then calculated by averaging the samples generated from a final set of iterations (e.g., the samples from the last 200 iterations). In the case of the discrete *α*_*gf*_ parameters, we select the value (either −1, 0, or 1) that has the highest frequency. The parameter estimates from this stochastic EM approach are then input as the initial starting values of our MCMC sampler. We choose to run a number of MCMC chains (in parallel) and implement the stochastic EM approach individually for each chain to produce different initial starting values. For final parameter inference, the lowest performing chains (i.e., the chains with the lowest marginal likelihoods) are discarded from analysis.

### Network inference

The association level network structure $\boldsymbol {\tilde {N}}=\left \{\tilde {n}_{gg'}\right \}_{\left (g,g'\right)}$ between genes is obtained by analyzing the posterior of the correlation matrix defined in Eq. (2). For each (*g*,*g*^′^) element in the correlation matrix, *M* samples are used to calculate the posterior mean $\widehat {\rho }_{gg'} = \frac {1}{M} {\sum \nolimits }_{m=1}^{M} \rho _{gg'}^{~(m)}.$ This estimate provides a quantifiable value of association between genes *g* and *g*^′^.

Since we are working in the Bayesian paradigm, we can examine the CI of the posterior to determine whether or not genes *g* and *g*^′^ are associated with one another. By choosing an appropriate level of significance *α*^∗^, two genes have a significant association when zero is excluded from the 100(1−*α*^∗^)*%* CI. A second method to determine significant associations from the posterior samples of $\rho _{gg'}\phantom {\dot {i}\!}$ is to find the smallest 100(1−a^∗^)*%* CI that includes 0. The corresponding a^∗^ value would indicate the proportion of the posterior distribution outside of the smallest CI that includes 0. Hence, we can think of a^∗^ as an approximate “*p*-value” that can be used to rank correlations by significance.

## Supplementary information

**Additional file 1** PDF file consisting of supplementary figures (S1 - S4).

**Additional file 2** Excel file containing lists of gene names and significant GO terms from the real data analyses.

## Data Availability

The datasets supporting the conclusions of this article are available in the GEO database repository, under accession numbers GSE60361 and GSE90975 (https://www.ncbi.nlm.nih.gov/geo). The R package for our HBFM model is available at: https://github.com/mnsekula/hbfm **Project name:** HBFM **Operating system(s):** Platform independent **Programming language:** R **License:** ≥ GPL version 2 **Any restrictions to use by non-academics:** None

## Abbreviations

AUC: Area under the operating characteristic curve
BN: Bayesian networks
CI: Credible interval
DIC: Deviance Information Criterion
FDR: False discovery rate
GCNs: Gene co-expression networks
GEO: Gene Expression Omnibus
GIG: Generalized inverse Gaussian
GO: Gene ontology
HBFM: Hierarchical Bayesian factor model
MBSC: Mouse brain single-cell
MCMC: Markov chain Monte Carlo
MMC: Mouse microglia cell
NORTA: Normal to anything
PCORR: Partial correlation
PPDs: Posterior predictive datasets
scRNA-seq: Single-cell RNA-sequencing
TPR: True positive rate
