# Supplementary material for: A sparse Bayesian factor model for the construction of gene co-expression networks from single-cell RNA sequencing count data
Source: BMC Bioinformatics. 2020 Aug 18;21:361. doi: 10.1186/s12859-020-03707-y (PMC7437941; doi:10.1186/s12859-020-03707-y)
Supplement: Supplementary file 1 — Additional file 1 PDF file consisting of supplementary figures (S1 - S4). [file 12859_2020_3707_MOESM1_ESM.pdf]

Additional file 1 for "A sparse Bayesian factor model for the construction of gene co-expression networks from single-cell RNA sequencing count data"

Michael Sekula[1], Jeremy Gaskins[1], Susmita Datta[2]

[1] Department of Bioinformatics and Biostatistics, University of Louisville, KY 40202, USA

[2] Department of Biostatistics, University of Florida, FL 32610, USA

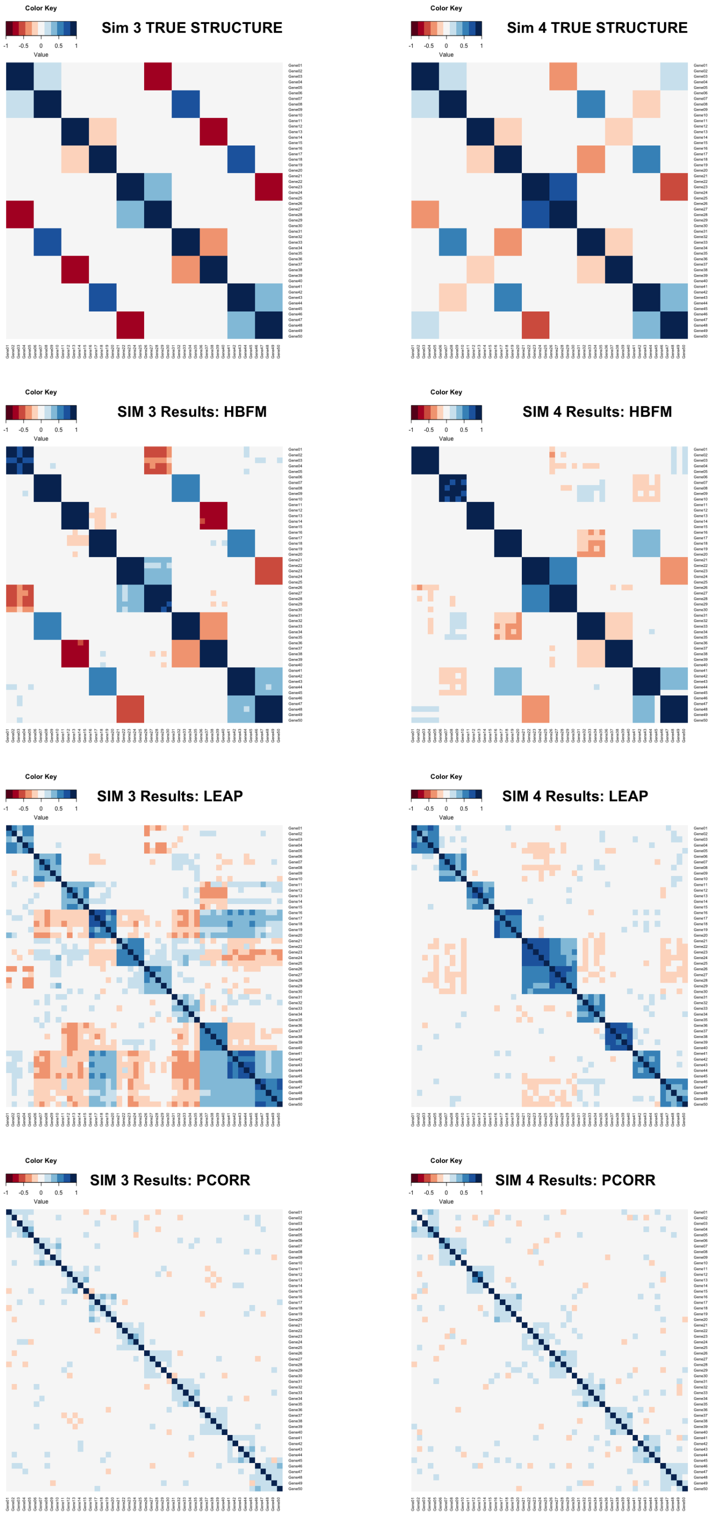

Figure S1: Heatmaps of the "true" correlation structure in Sim 3 ($F = 10, N = 500$) and Sim 4 ($F = 15, N = 500$) and the correlation structures estimated by HBFM, LEAP, and PCORR.

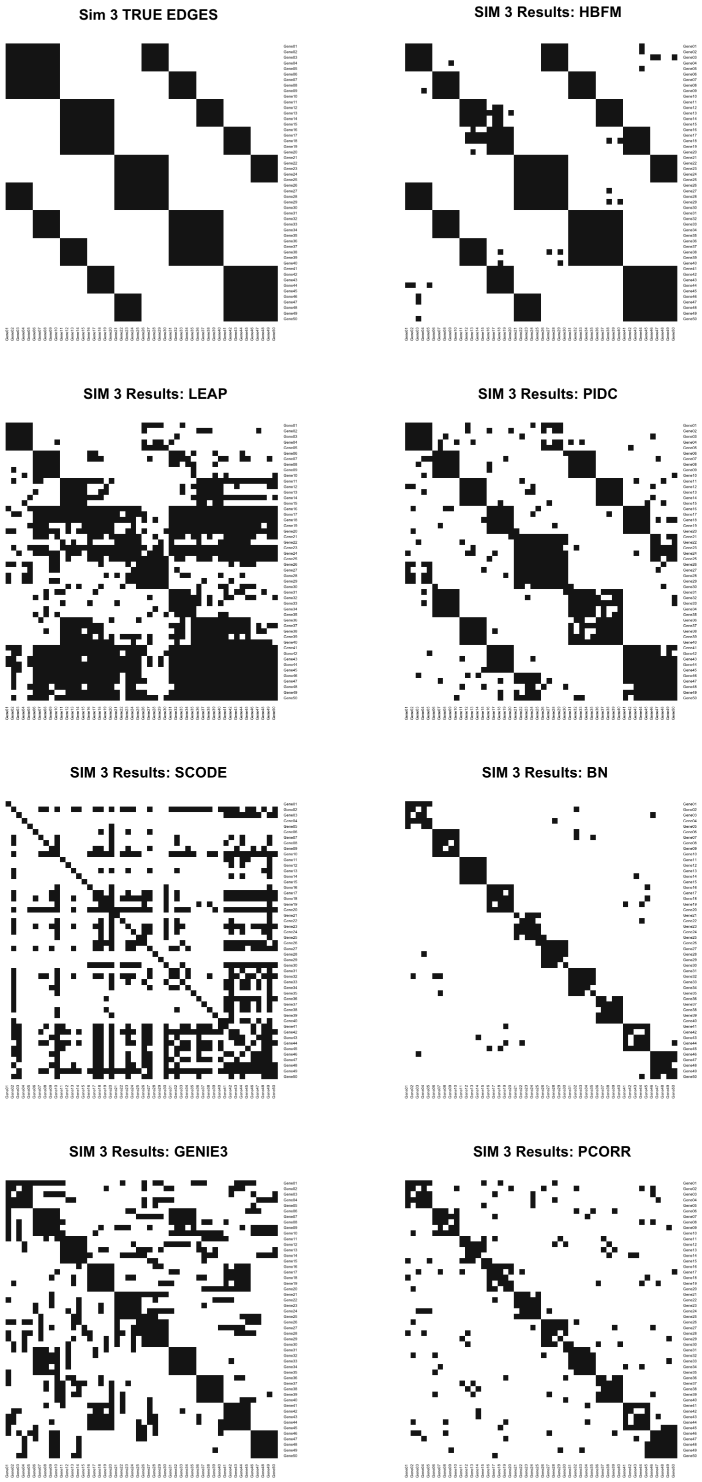

Figure S2: Heatmaps of the "true" network structure in Sim 3 (top left) and the network structures estimated by all network methods. The diagonal has been shaded in each figure to serve as a reference point. The shaded cells (other than the diagonal) indicate significant gene-gene associations. For the networks estimated by PIDC, SCODE, and GENIE3, the number of edges have been fixed to match the number of edges estimated by HBFM.

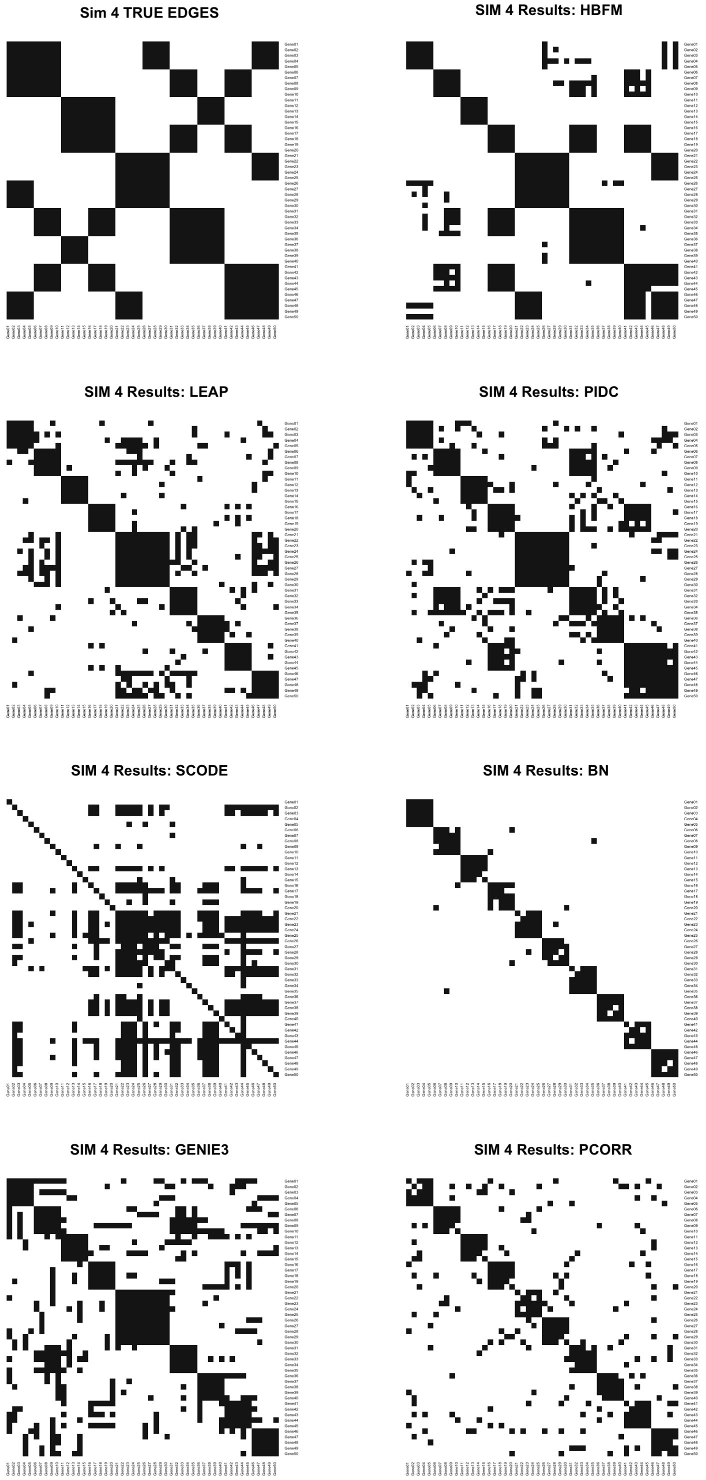

Figure S3: Heatmaps of the "true" network structure in Sim 4 (top left) and the network structures estimated by all network methods. The diagonal has been shaded in each figure to serve as a reference point. The shaded cells (other than the diagonal) indicate significant gene-gene associations. For the networks estimated by PIDC, SCODE, and GENIE3, the number of edges have been fixed to match the number of edges estimated by HBFM.

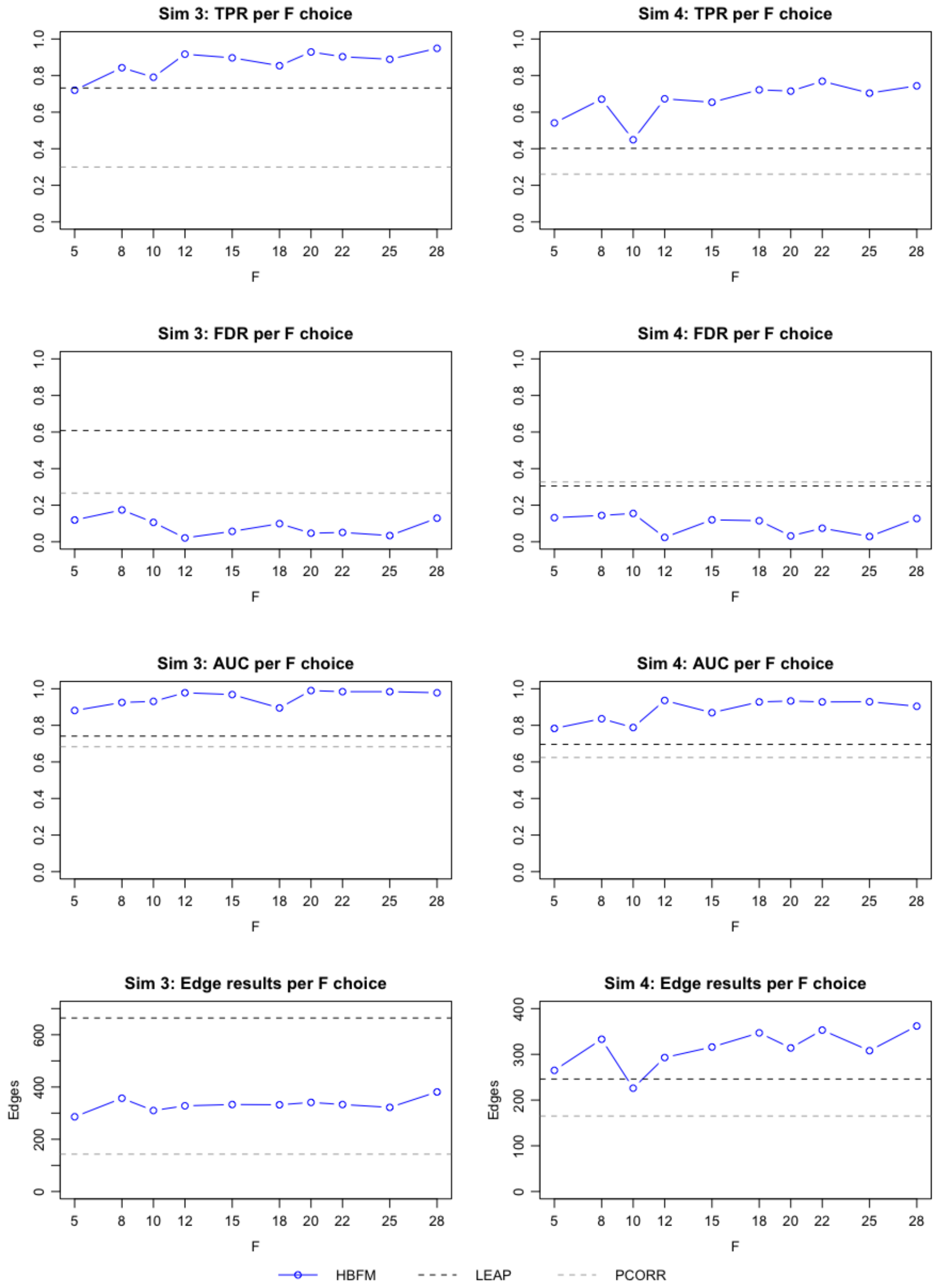

Figure S4: Performance measures for HBFM with different numbers of factors (F) for Sim 3 and Sim 4. The dotted lines represent the performance measures for LEAP and PCORR. Both LEAP and PCORR are correlation-based network methods and are the most similar to our proposed methodology.
